# *Vacciniummotuoense* (Ericaceae), a new species from Xizang, China

**DOI:** 10.3897/phytokeys.181.71522

**Published:** 2021-09-10

**Authors:** Yi-Hua Tong, Wang-Lin Zhao, Bing-Mou Wang, En-De Liu, Jie Cai, Yong-Jie Guo

**Affiliations:** 1 Key Laboratory of Plant Resources Conservation and Sustainable Utilization & Key Laboratory of Digital Botanical Garden of Guangdong Province, South China Botanical Garden, Chinese Academy of Sciences, Guangzhou, 510650, China; 2 Center of Conservation Biology, Core Botanical Gardens, Chinese Academy of Sciences, Guangzhou, 510650, China; 3 Motuo Observation and Research Center for Earth Landscape and Earth System, Chinese Academy of Sciences, Linzhi, 860712, China; 4 Panyu Central Hospital, Guangzhou, 511402, China; 5 CAS Key Laboratory for Plant Diversity and Biogeography of East Asia, Kunming Institute of Botany, Chinese Academy of Sciences, Kunming 650201, Yunnan, China; 6 Germplasm Bank of Wild Species, Kunming Institute of Botany, Chinese Academy of Sciences, Kunming, Yunnan 650201, China; 7 University of Chinese Academy of Sciences, Beijing100049, China

**Keywords:** Morphology, new species, *
Vaccinium dunalianum
*

## Abstract

*Vacciniummotuoense* (Ericaceae), a new species from Motuo County, Xizang Autonomous Region, China is described and illustrated. This new species belongs to Vacciniumsect.Calcicolus and is morphologically most similar to *V.dunalianum*, but differs in having yellowish-brown tomentose young branches, petioles and inflorescence rachis, leaf blades with 2–3(–4) pairs of secondary veins, usually all basal and with fine veins impressed adaxially and urceolate to spherical corollas.

## Introduction

The genus *Vaccinium* L. (Ericaceae), with about 450–500 species distributed worldwide, is the largest genus of the blueberry tribe or Vaccinieae Rchb. (Fang 1991; Fang and Stevens 2005; Vander Kloet and Dickinson 2009). In China, 95 species of *Vaccinium* are recorded, including the recently published *V.damingshanense* Y. H. Tong & N. H. Xia and *V.napoense* Y. H. Tong & N. H. Xia from Guangxi and *V.zhangzhouense* Y. H. Tong et al. from Fujian (Tong and Xia 2015; Tong et al. 2020, 2021). There are 15 species of *Vaccinium* recorded from Xizang Autonomous Region, most of which occur in the south to south-eastern part of that Province (Fang 1986).

During work on a revision of *Vaccinium* species from China, we observed that several specimens, identified as V.dunalianumvar.urophyllum Rehder & E. H. Wilson from Motuo County, southeast Xizang, differ substantially from those from other places in China in the leaf blade with relatively long caudate apex and the yellowish-brown tomentose inflorescence rachis. Thus, we conducted two field trips to collect fresh flowering and fruiting material for further study. We found that, in addition to the difference in the indumentum on the inflorescence rachis noted by Fang (1991: 95), the population of V.dunalianumvar.urophyllum from Motuo County could be further distinguished from other populations of that variety and, even, the entire species *V.dunalianum* Wight by the shape and venation of the leaf blade and the shape of the corolla. Thus, we concluded that the population of V.dunalianumvar.urophyllum from Motuo represents a new species, described below.

## Materials and methods

Fruiting and flowering material was collected from Motuo County, Xizang Autonomous Region, China during two field trips in January 2020 and May 2021. Descriptions were based on both living and dried collections, which were deposited at the Herbaria of the Institute of Botany, Chinese Academy of Sciences (**PE**), Kunming Institute of Botany, Chinese Academy of Sciences (**KUN**) and South China Botanical Garden, Chinese Academy of Sciences (**IBSC**). Measurements were performed with a ruler and small plant parts were observed and measured under a stereomicroscope (Mshot-MZ101).

## Taxonomic treatment

### Vaccinium motuoense

Taxon classificationPlantaeEricalesEricaceae

Y.H. Tong & Y.J. Guo sp. nov.

ECE1BCA8-B9A7-54CC-8D8E-AA86FFB2C034

urn:lsid:ipni.org:names:77219644-1

Figures 1 , 2

#### Type.

China. Xizang Autonomous Region: Motuo County, Km 80 on Zhamo Road, epiphytic on trees in evergreen broad-leaved forest, 29°40'59.9"N, 95°30'6.3"E, 2191 m a.s.l., 28 May 2021 (fl.), *Z. Liu & W. L. Zhao TYH-2523* (holotype: IBSC, isotypes: IBSC, KUN).

**Figure 1.**
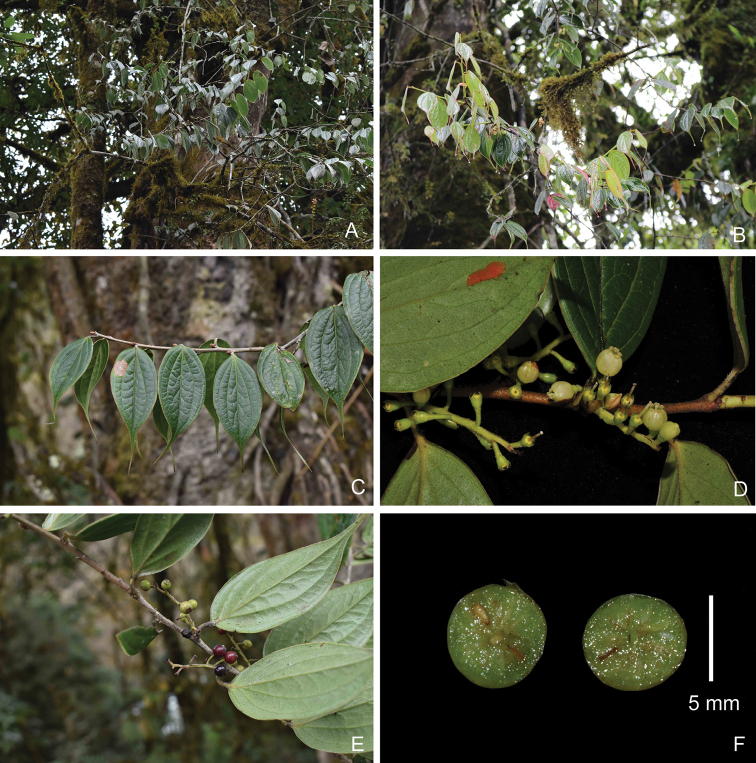
*Vacciniummotuoense* sp. nov. **A, B** habit **C** sterile branch with leaves **D** inflorescence **E** fruiting branch **F** fruits, cross section **A, C, E** and **F** taken by Y. H. Tong **B** by W. L. Zhao and **D** by J. Cai.

**Figure 2.**
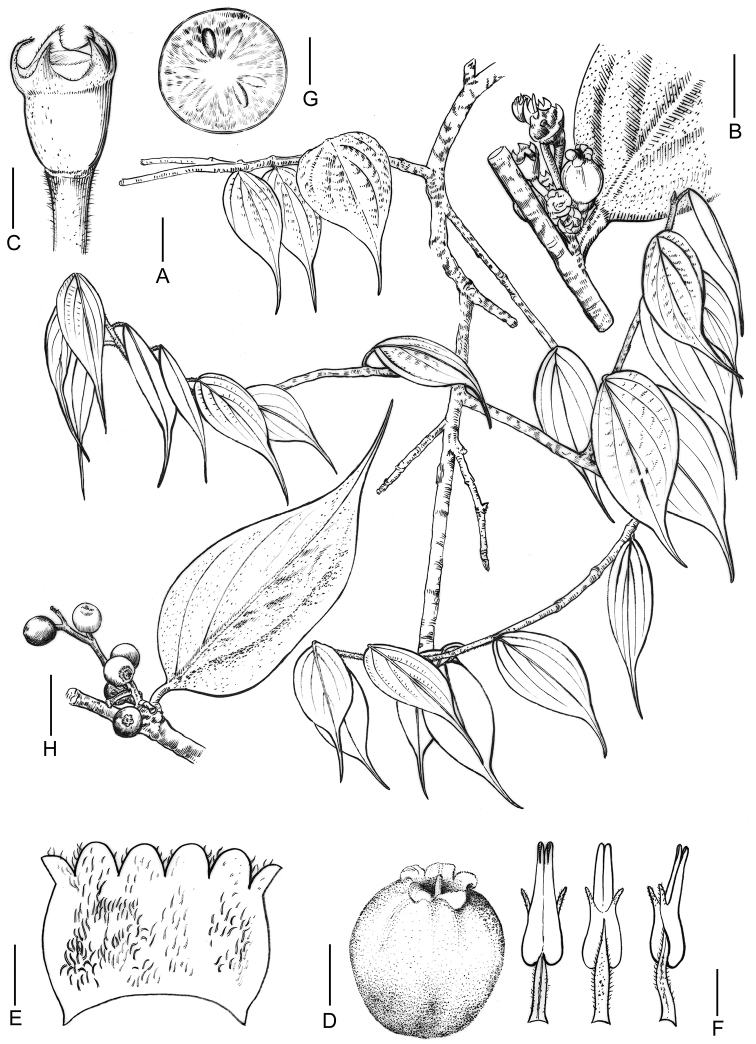
*Vacciniummotuoense* sp. nov. **A** sterile branches with leaves **B** inflorescence **C** calyx **D** corolla **E** opened corolla, showing pubescent internal surface **F** stamens, adaxial, abaxial and lateral view **G** transection of fruit **H** infructescence (Drawn by Ding-Han Cui). Scale bars: 2 cm (**A, B, H**); 5 mm (**C, G**); 2 mm (**D, E**); 1 mm (**F**).

#### Diagnosis.

Similar to *V.dunalianum* Wight, especially V.dunalianumvar.urophyllum Rehder & E. H. Wilson, in the hairy young branches, the shape, size and indumentum of leaf blade and the axillary racemose inflorescence, but distinguished by the yellowish-brown tomentose (vs. glabrous or brownish-white pubescent) young branches, petioles and inflorescence rachis, leaf blades with 2–3(–4) pairs (vs. 4–5) of secondary veins, usually all basal (vs. only lowermost 2–3 pairs basal), with veins impressed adaxially (vs. plane or slightly raised) and flowers with urceolate to spherical corollas (vs. broadly campanulate) (Table 1).

**Table 1.** Morphological comparison of *Vacciniummotuoense* and *V.dunalianum*.

Characters	*V.motuoense* sp. nov.	* V. dunalianum *
Young branches	Yellowish-brown tomentose	Glabrous or brownish-white pubescent
Petiole	Yellowish-brown tomentose	Glabrous or brownish-white pubescent
Leaf blade shape	Ovate or ovate-elliptic	Elliptic, oblong, oblong-lanceolate or ovate
Secondary veins	2–3(–4) pairs, usually all basal, with fine veins impressed adaxially	4–5 pairs, only lowermost 2–3 pairs basal, with fine veins plane or slightly raised adaxially
Inflorescence rachis	Yellowish-brown tomentose	Glabrous
Corolla shape	Urceolate to spherical	Broadly companulate
Filament	Puberulous and pubescent adaxially, slightly puberulous abaxially	Glabrous or puberulous both sides

#### Description.

Evergreen shrubs, epiphytic on tree trunks, sprawling or hanging, stem 0.5–3 m long, without swollen basal tuber or root swellings. Young branches brownish, slightly angled, without lenticels, yellowish-brown tomentose, glabrescent and becoming greyish-white when older. Perennating buds dimorphic (floral perennating buds are at least twice the size of vegetative perennating buds). Leaves alternate; petiole flattened above, 0.8–1.2 cm long, 1.7–2.2 mm wide, yellowish-brown tomentose throughout, glabrescent; blade ovate or ovate-elliptic, 8.5–13.5 (including caudate apex) × 2.4–5.0 cm, leathery, abaxially with dense appressed black-glandular trichomes, brown-tomentose or -pubescent on veins that are near leaf base both sides, otherwise glabrous, base broadly cuneate to rounded, with one basal gland per side at the junction of leaf base and petiole, margin flat or slightly revolute when dry, entire, apex long caudate 2–4 cm long; veins impressed adaxially, more so when dry, raised abaxially, secondary veins 2–3(–4) per side, usually all basal, short cross-veins (tertiary veins) between the secondaries prominent, nearly transverse and paralleled. Inflorescence racemose, usually axillary on biennial branches, 7–16-flowered. Peduncle usually very short, inflorescence rachis pale green, 0.7–3 cm long, yellowish-brown tomentose, glabrescent when fruiting; bracts pale green, obovate, cucullate, 6–8 × 4–6 mm, abaxially yellowish-brown tomentose or pubescent, adaxially glabrous, margin ciliate, caducous; bracteoles 2, inserted at base of pedicel, pale green, linear, 5–6 × ca. 0.2 mm, ciliate, caducous. Pedicel pale green, 2.5–4.5 mm long, yellowish-brown tomentose or pubescent or nearly glabrous, articulate with the hypanthium. Hypanthium green, cupuliform, 1.5–2.0 × 1.2–1.5 mm, glabrous; calyx limb lobed nearly to base, lobes 5, green, triangular, ca. 1.5 × 1 mm, apex acuminate, margin ciliate, with denser and longer cilia at apex. Corolla white or virescent, sometimes tinged with red on angles when young, urceolate to spherical, slightly angled when young, 4.5–6 × ca. 4.5 mm, glabrous outside, pubescent inside, 5-lobed; lobes ovate-triangular, reflexed, ca. 1 × 1.2 mm. Stamens 10, 4–4.5 mm long; filaments flat, slightly S-shaped, 1.5–2.0 mm long, puberulous and pubescent adaxially, slightly puberulous abaxially; anthers 3.0–3.5 mm long, thecae 1.5–1.7 mm long, tubules narrower than the thecae, 1.5–1.8 mm long, each with an oblique pore 0.7–1.0 mm long, 0.2–0.3 mm in diam.; spurs 2, borne at abaxial base of tubules, 0.8–1 mm long, echinate. Disc yellowish, annular, glabrous; style cylindrical, slightly angled in sicco, 5.0–5.2 mm long, glabrous, stigma punctate; ovary pseudo-10-locular, each locule with 8–10 ovules. Fruiting pedicel 4–11 mm long, expanded at apex; berry green when young, turning dark red later and finally dark purple at maturity, globose, 4–6.5 mm in diam., glabrous, with persistent calyx lobes appressed at apex. Seeds ovoid, 1.2–1.5 × 0.8–1 mm, testa brownish, reticulate, soft.

#### Etymology.

The species epithet is named after the type locality, Motuo County.

#### Vernacular name.

墨脱越橘 (Chinese pinyin: mò tuō yuè jú).

#### Distribution and habitat.

This species is currently known only from Motuo County, Xizang, China. It grows on trees in evergreen broad-leaved forests at elevations of 1600–2300 m.

#### Conservation status.

*Vacciniummotuoense* is common in the forests of Motuo County within an area over 3000 km^2^ and the whole area is under the protection of Yarlung Zangbo Grand Canyon National Nature Reserve. The threat risk seems to be low because it is not economically valuable and the conservation condition of the Reserve is good. Thus, it is assigned a status of ‘Least Concern’ (LC), following the IUCN Red List Categories and Criteria (IUCN Standards and Petitions Committee 2019).

#### Phenology.

Flowering in April-May and fruiting in October-March.

#### Additional specimens examined (paratypes).

China. Xizang, Autonomous Region, Motuo County: 3.5 km away from Bolonggong to Km 52 on Zhamo Road, 31 May 2013 (fl.), *J. Cai, E. D. Liu & Y. J. Guo 13CS7683* (KUN); Km 80 on Zhamo Road, 5 January 2020 (fr.), *Y. H. Tong & B. M. Wang TYH-2381* (IBSC); ibid. 12 January 1983 (fr.), *B. S. Li & S. Z. Cheng 02486* (KUN); Beibeng Xiang, Buqiong Hu, 11 November 1992 (fr.), *H. Sun, Z. K. Zhou & H. Y. Yu ETM-1145* (KUN); Beibeng Xiang, Gelin Cun, 20 May 1983 (fl.), *B. S. Li, Z. C. Ni & S. Z. Cheng 03651* (KUN), 3 January 2020 (fr.); ibid. *Y. H. Tong & B. M. Wang TYH-2338* (IBSC); Beibeng Xiang, Xirang, Sangxingpeng, 26 April 1983 (fl.), *B. S. Li, Z. C. Ni & S. Z. Cheng 04317* (KUN); Beibeng Xiang, Xirang, Xideng Shan, 25 April 1983 (fl.), *B. S. Li, Z. C. Ni & S. Z. Cheng 04752* (KUN); ibid. 12 January 1983 (fr.), *B. S. Li, Z. C. Ni & S. Z. Cheng 02486* (KUN); Damu Xiang, 6 March 1993 (fr.), *H. Sun, Z. K. Zhou & H. Y. Yu ETM-4243* (KUN); Damu Xiang, Dachi Shan, 30 October 1982 (fr.), *B. S. Li & S. Z. Cheng 01600* (KUN, PE); Damu Xiang to Gedang Xiang, 13 March 1993 (fr.), *H. Sun, Z. K. Zhou & H. Y. Yu ETM-4562* (KUN); Gedang Xiang, 21 March 1993 (fr.), *H. Sun, Z. K. Zhou & H. Y. Yu ETM-4920* (KUN); without precise locality, without date, *H. Sun, Z. K. Zhou & H. Y. Yu ETM-2621* (KUN).

## Discussion

According to Vander Kloet and Dickinson’s infrageneric classification of *Vaccinium*, *V.medongense* fits well with the circumscription of V.sect.Calcicolus Kloet that is characterised by an evergreen habit, dimorphic perennating buds (i.e. floral perennating buds at least twice the size of vegetative perennating buds), racemose inflorescences with large caducous bracts, pseudo-10-locular ovary, berry with 2–5 seeds per locule and soft seed testa (Vander Kloet and Dickinson 2005, 2009). In Xizang, besides *V.dunalianum*, there are two other species from the same section, viz. *V.gaultheriifolium* (Griff.) Hook. f. ex C. B. Clarke and *V.glaucoalbum* Hook. f. ex C. B. Clarke. However, these two species have glabrous and glaucous abaxial surface of leaf blades with serrate margins and acute or acuminate apices and, thus, are easily distinguishable from *V.motuoense*. This new species is common in its area of distribution, usually growing together with other epiphytic plants on tree trunks covered with mosses, such as orchids, ferns and some other ericaceous species like *Agapetespraeclara* C. Marquand, *A.forrestii* W. E. Evans, *Vacciniumleucobotrys* (Nutt.) G. Nicholson, *V.retusum* Hook. f. ex C. B. Clarke and *V.kingdon-wardii* Sleumer.

## Supplementary Material

XML Treatment for Vaccinium motuoense
